# Supplementary material for: Quantification of porosity in extensively nanoporous thin films in contact with gases and liquids
Source: Nat Commun. 2019 Sep 27;10:4394. doi: 10.1038/s41467-019-12277-4 (PMC6765025; doi:10.1038/s41467-019-12277-4)
Supplement: Supplementary file 1 — Supplementary Information [file 41467_2019_12277_MOESM1_ESM.pdf]

Supplementary Information

Quantification of Porosity in Extensively Nanoporous Thin Films in Contact with Gases and Liquids

Shpigel et al.

Supplementary Note 1. QCM-D measurements in air and in contact with liquids

This paper presents a new methodology for the assessment of fine porous structures using non-invasive, non-destructive approach based on quartz crystal microbalance sensors with momentum dissipation sensing (QCM-D). The systems that we intend to explore are located on the surface of the thin quartz crystal sensors. The methodology is demonstrated herein by thin nanoporous films of Au-Zn alloys (deposited on the quartz crystals). Quantification of the porosity of structures using QCM-D can be based both on determination of the specific gravimetric density of porous measured in air and on the amount of liquid trapped in the porous structure. Thickness of the porous layer should be obtained by one of the complementary techniques.

In order to provide reliable measurements, it is necessary first of all to validate that the quality of the sensor is not deteriorated by the electrochemical processes that the system undergoes. Such validation can be done based on the intensity and the shape of the measured resonance peaks in both air and liquid phases. After careful washing of the porous sensor from solution residues and drying under vacuum, we have analyzed the resonance peaks (RP) response for the entire overtones range in air and under water. A compression between the RP of bare quartz crystals (QC) and representative porous structures located on their surfaces reveals that in both media the intensities and the peak widths of non-porous and the cycled sensors (namely, the QC and the layers deposited on them) are virtually the same, thus implying appropriate retention of the high quality of the QC based sensors that we use. Interestingly, for both bare and porous QC the dispersion of the normalized frequencies in air and water are different: while in air a gradual shift toward lower frequency values with increased overtone number was detected, less dispersed signals have been recognized under water. Such a behavior is ascribed to naturally existing flexural vibrational modes which shift the resonance frequency to more positive values. This phenomenon is more pronounced at lower overtones having larger vibration amplitude. As the sensor operates under liquids, the contribution of these non-tangential waves is suppressed by the pressure of the liquid expressed by more overtone independent response[1]

Supplementary Note 2. Porosity calculation using QCM-D operated in liquid phases

During recent years, the research opportunities and applications offered by the QCM-D techniques are continuously growing. The capability of QCM-D to measure simultaneously multiple overtones provides an effective way for monitoring of gravimetric, structural and mechanical changes in biological samples,

polymeric and ceramic coatings, as well as battery materials. At a first glance, it seems that detection of gravimetric changes by QCM doesn't require using of several oscillation modes, since a linear correlation between the accumulated mass and the frequency changes according to the Sauerbrey equation (Supplementary Equation 1) is used for that, see the main text[2]:

However, this equation can be used only for thin (i.e. with thickness smaller than the wavelength of sound), rigid and uniform coating, and the decision whether the coated film meets these requirements should be based on both frequency and dissipation (equivalent to the resonance width through $D = \frac{W}{f}$) signals recorded from several overtones. In general, only when identical overtone normalized frequency changes followed by negligible variation of the dissipation factor (relative to the dissipation value of the un-coated QC operated under similar conditions) are obtained, the coating can be considered as a 'Sauerbrey film'[3](i.e. its changes in mass can be precisely calculated by this equation). When ideally flat quartz sensor oscillates in a liquid environment both frequency and dissipation are affected by the fluid mass loading and its viscous damping. In this case, the $\Delta f/n$ and $\Delta W/n$ values are given by the Kanazawa equations[4]:

$$\frac{\Delta f}{n} = -f_0^{\frac{3}{2}} \frac{\sqrt{\rho_l \cdot \mu_l}}{n Z_q}; \; \frac{\Delta W}{n} = 2 f_0^{\frac{3}{2}} \frac{\sqrt{\rho_l \cdot \mu_l}}{n Z_q} \tag{1}$$

Where ρ_l, μ_l are the density and the viscosity of the liquid respectively, f_0 is the fundamental resonance frequency of the QC, and n is the overtone number. Z_q = 8.8×10^6 kg/(m^{2}s) is a constant value related to the acoustic wave impedance of AT-cut quartz crystal (i.e. relevant to the elastic modulus and density of the quartz). The penetration depth of the oscillation waves toward (and inside) the contacting liquid is given by Eq. (2) of the main text.

It is seen that the values of δ are affected by the nature of the fluid and varies with the overtone number. The situation is changed when the QC is coated by a non-flat layers (rough or porous) of a rigid morphology. In this case, the hydrodynamic forces acting between the layer structural features and the surrounding liquid may also contribute to the recorded *f* and *W* values[5]. Based on the fingerprint of the multi-harmonic *f* and *D* signals, the structural parameters of the film can be retrieved using appropriate hydrodynamic equations. Focusing on the characterization of porous films, one should take in account two morphological parameters of the deposited layers, namely the permeability length, ξ (structural factor linked to their porosity which describes the ability of a porous medium to permit fluids to flow through it) of the coating and its thickness – *h*. In the case of meso-porosity (and as well for micro-

porosity) it can be assumed that $\xi << \delta$. Under such circumstances, the liquid inside the porous layer can be treated as a non-movable filler that contribute only to the changes in frequency (rather than dissipation) and can be directly translated to mass change. The volume of the porous layer can be extracted from its density. In contrast, when $\xi >> \delta$ the liquid is free to move in the pores giving a rise to the hydrodynamic interactions which affect both f and W [6]. For such a situation, estimation of the total pore volume is not trivial since there is no direct way to separate the gravimetric contribution from the total response. It is worth noting that for some defined structures (like inter-connected porous layers) the porosity may be evaluated using Kozeny-Carman equation. A convenient way to recognize whether the inserted liquid can be considered as a trapped or a movable entity in porous coatings, is to plot the values of Δf and ΔW (both normalized by a constant factor of $f_0^2\rho$) versus the penetration depth of the contacting liquid. As it can be seen from Supplementary Figure 4, for the ideally flat sensor (bare QC) tested under water, the experimental value of Δf and ΔW are following the theoretical dashed lines (as predicted from the Kanazawa equation). After the performance of the alloying/dealloying cycles, the immersion of the porous sensor in water resulted in a negative frequency shift followed by only negligible changes in the resonance width. Impregnation by ethanol and hexane characterized by the different penetration depth values, was expressed by a similar frequency shift, implying that the penetration of the liquid into the pores is not dependent on the liquid nature but rather on the morphology of the coating.

Supplementary Note 3. Viscoelastic modeling

Complex frequency change defines Re and Im parts according to Supplementary Equation 2

$$\Delta f^* = \Delta f + i\frac{\Delta W}{2} \tag{2}$$

For a viscoelastic film in contact with a liquid phase, the complex frequency change is defined by Supplementary Equation 3[1]:

$$\left(\frac{\Delta f^*}{n}\right) = -\frac{f_0}{n}\frac{Z_f^*}{\pi Z_q}\frac{Z_f^*\tan(k_f^* d_f) - iZ_{liq}^*}{Z_f^* + iZ_{liq}^*\tan(k_f^* d_f)} \tag{3}$$

Here the wavenumber k_f* is defined by Supplementary Equation 4:

$$k_f^* = \frac{\omega}{Z_f^*} d_f \tag{4}$$

Where the angular velocity is $\omega = 2\pi n f_0$, d_f denotes thickness of the viscoelastic layer whereas the

acoustic load impedance Z_f^* and shear-wave impedance of liquid Z_{liq}^* are defined by Supplementary Equations (5) and (6):

$$Z_f^* = (\frac{\rho_f}{J_f})^{1/2} \tag{5}$$

$$Z_{liq}^* = (i\omega\rho_{liq}\eta_{liq})^{1/2}$$

(6)

ρ_{liq} and η_{liq} are density and shear viscosity of the liquid, ρ_{liq} is density of viscoelastic layer, whereas the complex shear modulus of the viscoelastic layer, G_f^* is the reciprocal of the complex compliance, J_f^*, defined by Supplementary Equation 7:

$$G_f^* = \frac{1}{J_f^*} \tag{7}$$

The complex shear modulus G_f^* is composed of storage and loss moduli, G_f' and G_f'' by Supplementary Equation 8:

$$G_f^* = G_f' + iG_f'' \tag{8}$$

Finally, the loss modulus is linked to the shear viscosity η_f by Supplementary Equation 9:

$$G_f'' = \omega\eta_f \tag{9}$$

The viscoelastic modeling was performed as was previously described in our papers and in the literature[7,8]. Equations (2)-(9) are implemented in the commercial software Qtools (3.1.25.604 from Biolin Sci., Sweden). By fitting the viscoelastic models to the experimental data, i.e. ΔD and $\Delta f/n$ changes, containing the following four parameters are evaluated: the layer density ρ_f, the thickness h_f, the elastic shear storage modulus G', and the shear loss modulus G'', which is linked to the layer shear viscosity by Eq. (9). Four physically different samples were measured. Error analysis is presented in SI Section 6.

Supplementary Note 4. Grazing-Incidence Small-Angle X-ray Scattering (GISAXS) measurements.

As an additional complementary analysis, the porosity of the sensor (i.e. the QC + the porous layer deposited on it) was quantified using a GISAXS technique. In this method, the porosity is estimated based on the ratio of critical angles obtained for the porous film and for the non-porous bulk Au-Zn alloy. While for both previous techniques it is necessary to perform an independent measurement of the layer thickness, here the porosity can be calculated by finding the critical angles for both systems. The critical angle is related to the film density by the following equation[9] :

$$\alpha_c = \sqrt{\frac{\rho N_a r_e \lambda^2 \sum_i c_i f_i}{\pi \sum_i c_i M_i}} \quad (10)$$

where ρ is material density [g/cm^3], N_a is the Avogadro constant, r_e is the classical electron radius (2.8179403 x 10^{-15} m), M is the molar mass [g/mol], λ is the scattering wavelength (0.154×10^{-6} mm in our experiment), f is the atomic scattering factor and c is the stoichiometric number in the chemical formula or the mole concentration in the multi-component system.

The critical angle (α_c) of a non-porous bulk alloy (0.83/0.17 Au-Zn) was calculated from Supplementary Equation 11, see Supplementary Table 3a). The experimental critical angle was extracted from the location of Yoneda peak. As in can be seen in Supplementary figure 5, the maximal intensity of the off-specular diffuse scattering profile (i.e. the Yoneda peak) was observed for at 0.92 incident angle corresponding to 0.395 degrees of Yoneda angles. The ratio between the theoretical $\alpha_{c,n}$ and the measured angle $\alpha_{c,p}$ can be translated into porosity using:

$$\phi = 1 - \left(\frac{\alpha_{c,p}}{\alpha_{c,n}}\right)^2 \quad (11)$$

Based on this ratio, the calculated porosity is 36.8±1.8%, in a good agreement with the previous independent measurements.

Supplementary Note 5. Measurements of standard errors and errors propagation for calculated porosity values.

a. QCM-D measurements in air and thickness measurements by AFM

The QCM-D instrument has tremendously high precision in measurements of resonance frequency. 5 MHz crystal can be measured with an absolute error of only ±0.1 Hz. However, this high precision can be observed during measurements of the complex resonance frequency changes (rather than the absolute values of frequencies) provided that all the numerous factors affecting the stability of the frequency measurements (temperature, pressure, remaining static stress in the crystal because of the use of o-rings connecting the oscillation crystals with their holder of the measurement cell) are kept constant.

For the determination of the absolute values of nano-porosity of the Au-Zn alloys (selected as a suitable example for this study) by QCM-D in air, the crystals are assembled/disassembled in the measurement cells twice whereas determination of nanoporosity in liquids implies a triple assembling/disassembling of the crystals.

The first assembling is performed to measure QCM-D response of neat flat Au-covered crystal. Then the cell is disassembled and the crystal is inserted into an electrochemical cell in which the Au-Zn nano-porous film are fabricated at elevated temperatures. Carefully washed and dried crystals with Au-Zn nanoporous layers are assembled into QCM-D cells for measurements in air. The mass density of the alloy is calculated by comparison of masses of the porous alloy and of the mass of the Au on the neat quartz crystal taking into account that the amount of Au is kept constant whereas an additional constant amount of Zn remains in the alloy.

b. QCM-D measurements in air and thickness measurements by AFM

When measurements are performed in liquids, the crystal with the Au-Zn alloy on its top is disassembled from the cell, and is immersed into liquid in a separate cell. It is first evacuated in order to remove air from the pores and then is filled by liquid under vacuum (this is similar to a standard procedure of impregnation of composite porous battery electrodes with electrolyte solutions). Impregnation of porous Au-Zn alloys by a simple immersion into liquid without use of vacuum can result in incompleted impregnation because of the air remaining in the pores.

The absolute standard error of a neat flat crystal in air 4 times assembled/disassembled in QCM-D cell was ± 8 Hz (see Supplementary Figure S6a). Practically the same error was observed for assembled/disassembled crystals with nanoporous Au-Zn alloys (not shown). However, when 4 different samples with Au-Zn alloy were characterized in air, the absolute standard error of frequency measurement increased by a factor of two, i.e. up to ± 16.4 Hz (see Supplementary Figure 6b). Taking into account that the formation of Au-Zn alloy resulted in frequency decrease in air by 1.61 kHz, the relative error of frequency measurements of four different samples was ± 1 %.

Below we present a detailed scheme of the estimation of errors in measured thickness and frequency of the Au-Zn alloy in air. Four different samples had the following measured by AFM thicknesses: 180, 173, 188 and 179 nm. The calculated standard error is ±3.08 nm, so that the mean thickness with error is 180±3.08 nm.

The mass density of the same four samples (calculated from the measured frequency changes) were 175.95, 165.30, 169.73, and 170.02. Thus the mean mass density with standard error was 170.00±1.97 $\mu g/cm^2$.

Error propagation for the values of porosity were calculated using Eq. (3) of the main text:

$$\phi = [1 - (m_p/h_p\rho_{np})]$$

The specific gravimetric density of the non-porous Au-Zn alloy was calculated using the additivity rule for the components as indicated in the main text. The calculated value ρ_{np}=15.01 g/cm^3 was kept constant for all 4 porous samples. Using this formulae and the standard errors indicated above, we calculated the minimal and maximal values of porosity, from which we obtained the mean value of porosity with error: 37.04 ± 1.81 %.

For calculation of porosity value by weighing of the trapped liquid (see Fig. 4) we used Eq. (4) of the main text:

$$\phi = m_{liq} / \rho_{liq} h_p$$

The mean thickness of the porous Au-Zn alloy is 180±3.08 nm as in the previous calculation of porosity in air whereas mean mass density of trapped liquid with error was found to be 6.585±0.33 μg/cm^2 (relative error in weighing of trapped liquid is much higher than that of weighing of the porous Au-Zn solid in air because the latter is much larger than the former one). The calculation of the minimal and maximal values of porosity results in its mean value with error: 36.60±2.45 %.

c. Errors in calculation of viscoelastic characteristics of porous Au-Zn alloys

One and the same viscoelastic model (as indicated in SI Section 4) was applied for treatment of QCM-D characteristics of 4 different samples of the nanoporous alloy. Mean storage and loss moduli with errors were found to be 20.00±1.83 and 24.90±2.45, respectively.

The calculated mean effective gravimetric density of nanoporous alloys filled with liquid was 9.80±0.33 g/cm^3. The amount of trapped liquid was calculated above (6.585±0.33 g/cm^2) which was translated into the partial gravimetric density of liquid in the alloy equal to 0.366±0.018 g/cm^3. Hence the calculated gravimetric density of porous Au-Zn alloy is 9.43±0.33 g/cm^3. This leads to the mean porosity of the alloys with error: 37.15±2.18 %.

d. Errors in GISAXS calculations

As noted in the previous section, the porosity value was evaluated from the ratio between the theoretical critical angel (calculated according to the formed Au/Zn ratio), and the measured critical angle found from the GISAXS analyses carried out for the porous sample (given by Eq. (11)). The experimental value of the critical angel, [degrees], found from difference between the scattering vector measured at the location of the Yoneda peak and the scattering vector measured at the place of the specular beam (h_Y and h_f respectively), are given by the following formula:

$$\alpha_{c,p} = 2\theta_Y - \theta_f = 2\frac{180}{\pi}\left(\frac{h_Y\lambda}{4\pi}\right) - \frac{180}{\pi}\left(\frac{h_f\lambda}{4\pi}\right) \quad (12)$$

(13)

$$\alpha_{c,p} = 2\frac{180}{\pi}arcsin(\frac{h_Y\lambda}{4\pi}) - \frac{180}{\pi}arcsin(\frac{h_f\lambda}{4\pi}) \sim 2\frac{180}{\pi}(\frac{h_Y\lambda}{4\pi}) - \frac{180}{\pi}(\frac{h_f\lambda}{4\pi})$$

Where $2\theta_Y$ and $2\theta_f$ are the angels of the Yoneda and the specular peaks maximum positions respectively and λ is the scattering wavelength. For relative error estimation, the measurement has been performed several times and the maximum variation of the scattering vectors was found to be 0.002Δh ~ 0.002* h. Considering this estimated value, the relative critical angle error for the porous film $\theta_{c,p}$ is:

(14)

$$\frac{\Delta\alpha_{c,p}}{\alpha_{c,p}} \sim \frac{0.002 \cdot (2h_{Yoneda} + h_f)}{2 \cdot h_{Yoneda} - h_f} \sim \frac{0.002 \cdot (2 \cdot 1.155 + 1.755)}{2 \cdot 1.155 - 1.755} \sim 0.015$$

The absolute error for the measured porosity Pp is given by:

(15)

$$Pp\left(\frac{\Delta\alpha_{c,p}}{\alpha_{c,p}}\right) = \left[1 - \left[\frac{0.39\left(1 + \frac{\Delta\alpha_{c,p}}{\alpha_{c,p}}\right)}{0.498}\right]^2\right] * 100 - \left[1 - \left(\frac{0.39}{0.498}\right)^2\right] * 100 \sim 1.8\%$$

Hence, the porosity was determined to be (36.83±1.80)%

e. Errors in RBS calculations

Here we considered the number of measured counts as the main error source. The precision of our measurement was found to by ±1.2% which is in a good agreement with the literature reported data[10,11]. As noted previously, the calculated standard error for the thickness is ±3.08 nm, given a mean thickness of 180±3.08 nm.

Taking into account the sum of the both error values, the calculated mass density was determined to be 9.54±0.32 μg/cm^2, resulting in the porosity value (36.42±2.15) %.

Supplementary Figures

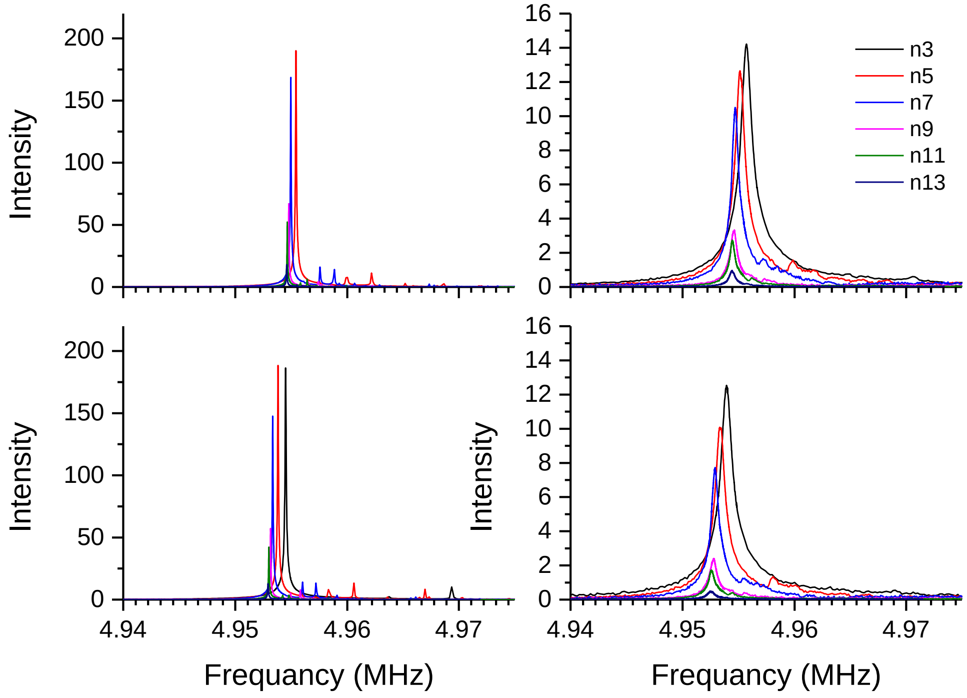

Supplementary Figure 1. Resonance peaks measured by QCM-D for thin porous Au-Zn alloy film fabricated on the QC surface. The normalized by the overtone order responses for 3rd to 13th harmonics recorded in air and in contact with water for the neat crystal before cycling (a, b, respectively) and for the quartz crystal containing porous alloy after 7 cycles (c, d, respectively).

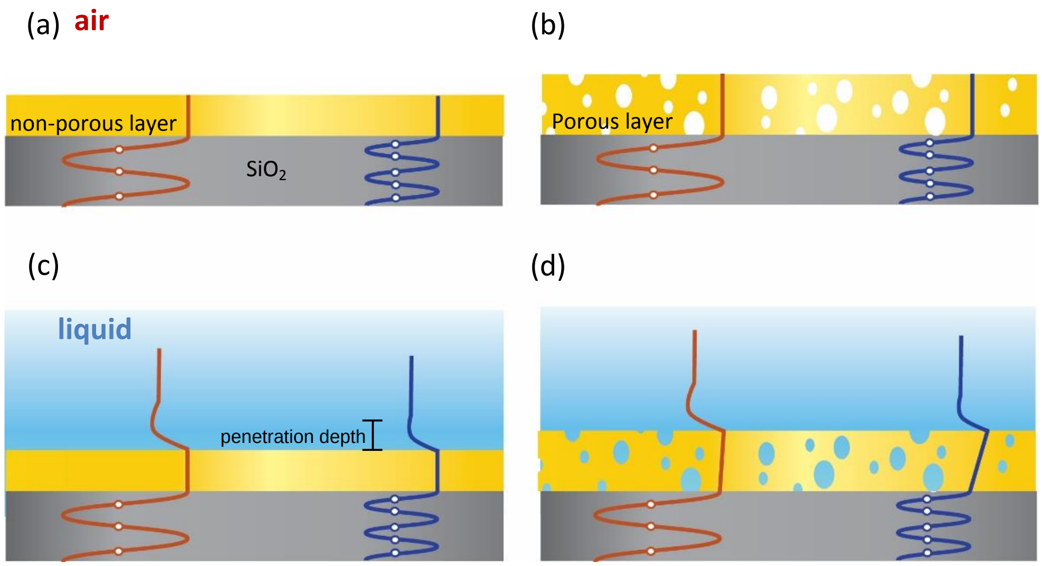

Supplementary Figure 2. Generation of acoustic shear waves by quartz crystal resonators (oscillation on 1st and 5th harmonics are shown) covered with a thin nonporous and nanoporous Au layer (a, c, and b, d respectively). The velocity profiles of the acoustic waves across the Au layer in contact with liquid phase are shown in panels (a, b) and (c, d), respectively. The penetration depth in liquid is indicated by vertical line. The sloping profile of the acoustic wave across the porous alloy (panel d) reflects its viscoelasticity on higher harmonics.

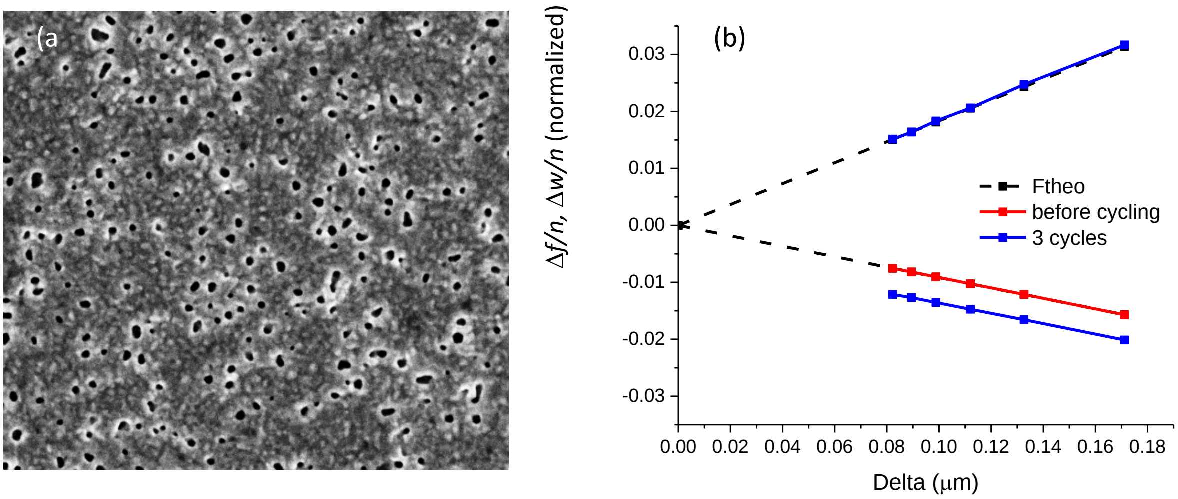

Supplementary Figure 3. SEM image (a), and the frequency and resonance width changes as a function of the penetration depth of the nanoporous Au-Zn layer in contact with water (b) as observed after the 3rd cycle.

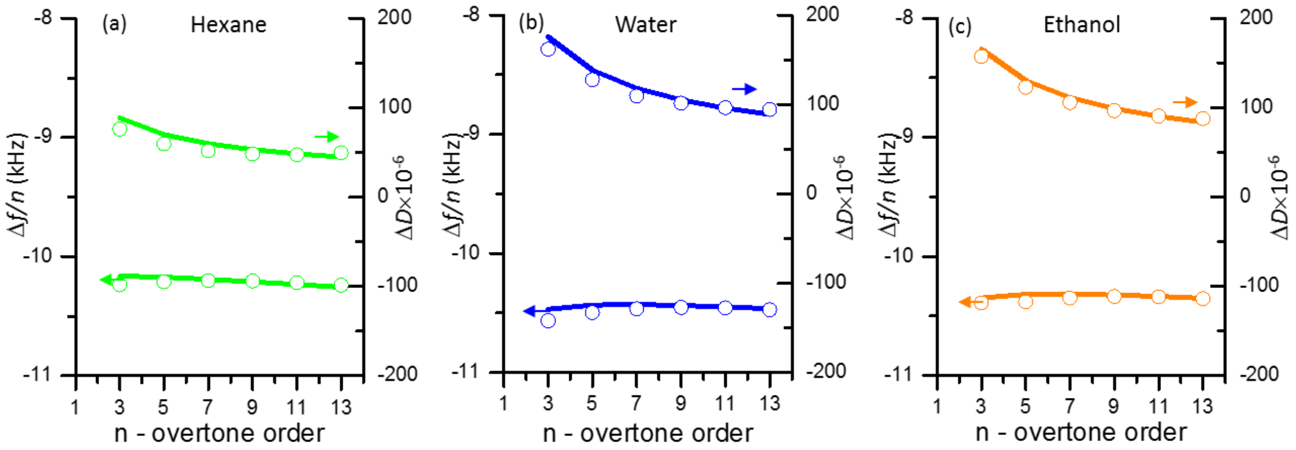

Supplementary Figure 4. Determination of porosity of Au coating onto quartz crystal in contact with liquid from QCM-D measurements and the related viscoelastic modeling. Frequency and dissipation changes (left and right ordinate axes, respectively) for the porous alloy immersed in hexane, water and ethanol (panels a, b and c, respectively). Open circles and solid lines denote the experimental points and fitting curves, respectively.

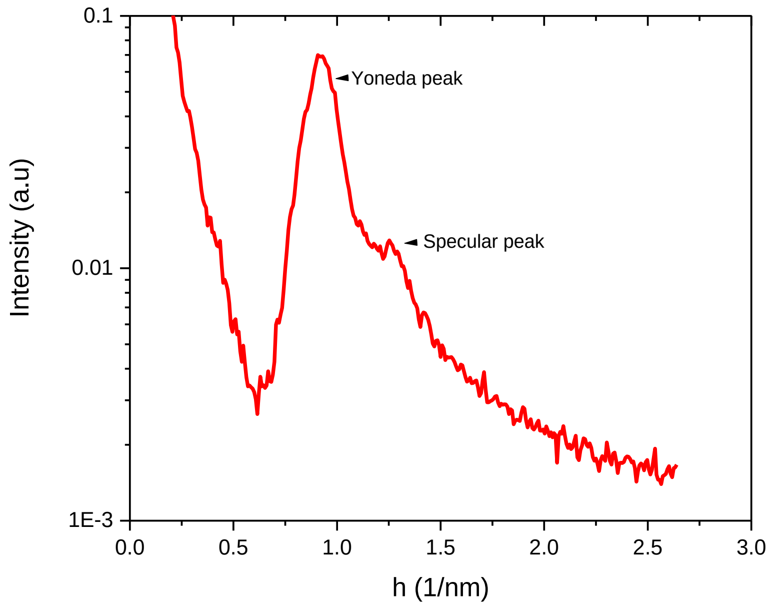

Supplementary Figure 5. A vertical profile of GISAXS for a QC covered by a porous alloy layer (denoted also as a porous sensor). The locations of the specular and Yoneda peaks indicated in the chart.

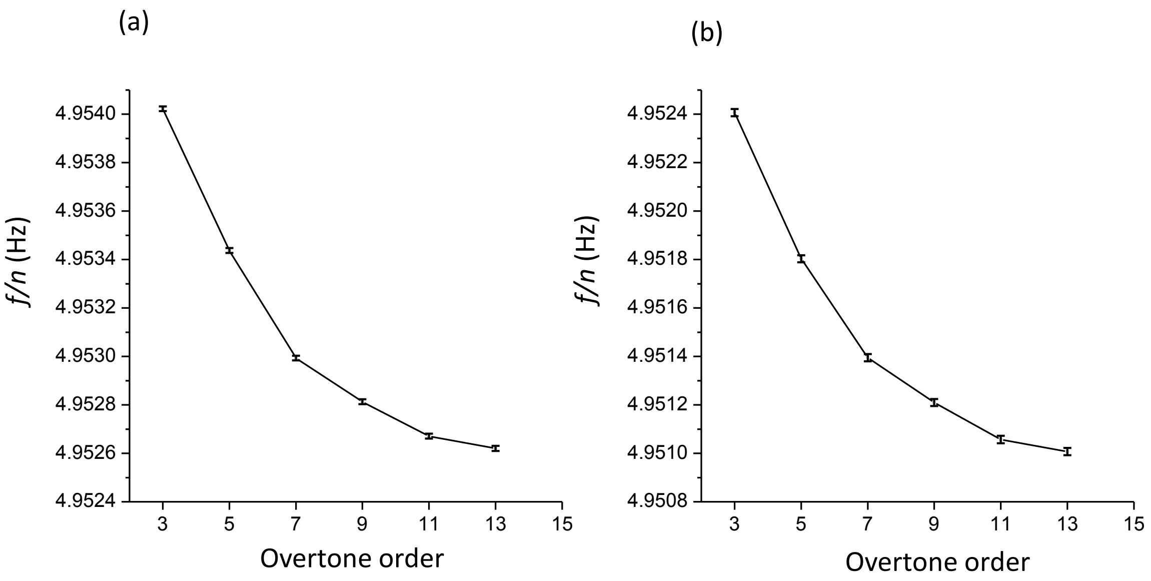

Supplementary Figure 6 . QCM-D measurements of absolute value of the resonance frequency of neat Au-coated crystal at all odd overtones from 3 to 13 in air (a). The crystal was assembled and disassembled 4 times. The standard error was found to be ± 8 Hz. QCM-D measurements of absolute values of resonance frequency of 4 different samples of Au-Zn nanoporous alloys in air (b); all 4 samples were prepared under identical conditions. The standard error was found in this case to be ± 16.4 Hz.

Supplementary Tables

Supplementary Table 1. Viscoelastic parameters and physical properties of semi-infinite Newtonian liquids in contact with porous Au-Zn layers used for fitting the related frequency and dissipation changes.

Properties	Hexane	Ethanol	Water
Fluid density (kg/m^3)	655	785	997
Fluid viscosity (kg/ms)	2.97×10^{-4}	1.00×10^{-3}	8.94×10^{-4}
Porous layer density (kg/m^3)	9800	9800	9800
Shear viscosity (kg/ms)	0.8	0.8	0.8
Storage modulus (Pa)	2.00×10^{7}	2.00×10^{7}	2.00×10^{7}
Layer thickness (nm)	181	181	181
Viscosity exponent	-0.85	-0.85	-0.85
Shear exponent	1.35	1.35	1.35

Supplementary Table 2. Elemental areal density (atom/cm^2) for the non-cycled sensor and for the porous QC as analyzed from the RBS spectra.

	Au	Cr	Zn
Before cycling	4.34×10^{17}	3.75×10^{16}	
After 7 cycles	4.37×10^{17}	4.05×10^{16}	2.66×10^{17}

Supplementary Table 3. Raw parameters used for calculation of critical angle for the non-porous Au/Zn

Material	Density, g/cm^3	c	M, g/mol	f	θ_c, Critical angle, degrees
Au	19.32	1	196.97	75.40	0.558
Zn	7.14	1	65.39	28.54	0.362
Au-Zn	15.01	1.656	154.44	57.75	0.497

Supplementary References

1. Johannsmann, D. *The Quartz Crystal Microbalance in Soft Matter Research*. *The Quartz Crystal Microbalance in Soft Matter Research* (2015). doi:10.1007/978-3-319-07836-6

2. Sauerbrey, G. Use of a quartz vibrator for weighing thin films on a microbalance. *Zeitschrift für Phys.* **155**, 206–222 (1959).

3. Johannsmann, D. *The Quartz Crystal Microbalance in Soft Matter Research*. (Springer International Publishing, 2015). doi:10.1007/978-3-319-07836-6

4. Keiji Kanazawa, K. & Gordon, J. G. The oscillation frequency of a quartz resonator in contact with liquid. *Anal. Chim. Acta* **175**, 99–105 (1985).

5. Levi, M. D. *et al.* In Situ Porous Structure Characterization of Electrodes for Energy Storage and Conversion by EQCM-D: a Review. *Electrochim. Acta* **232**, 271–284 (2017).

6. Shpigel, N. *et al.* Direct Assessment of Nanoconfined Water in 2D Ti_3C_2 Electrode Interspaces by a Surface Acoustic Technique. *J. Am. Chem. Soc.* jacs.8b04862 (2018). doi:10.1021/jacs.8b04862

7. Voinova, M. V., Rodahl, M., Jonson, M. & Kasemo, B. Viscoelastic Acoustic Response of Layered Polymer Films at Fluid-Solid Interfaces: Continuum Mechanics Approach. *Phys. Scr.* **59**, 391–396 (1999).

8. Dargel, V. *et al.* In situ real-time gravimetric and viscoelastic probing of surface films formation on lithium batteries electrodes. *Nat. Commun.* **8**, 1389 (2017).

9. Goh, T. K. & Wong, T. K. S. Investigation of structure, thermal and oxygen plasma stability of mesoporous methylsilsesquioxane films by X-ray reflectivity and small angle scattering. *Microelectron. Eng.* **75**, 330–343 (2004).

10. Colaux, J. L. & Jeynes, C. High accuracy traceable Rutherford backscattering spectrometry of ion implanted samples. *Anal. Methods* **6**, 120–129 (2014).

11. Jeynes, C., Jafri, Z. H., Webb, R. P., Kimber, A. C. & Ashwin, M. J. Accurate RBS Measurements of the Indium Content of InGaAs Thin Films. *Surf. Interface Anal.* **25**, 254–260 (1997).
